# Supplementary material for: Serum immunoglobulin M is associated with the severity of coronary artery disease in adults
Source: PeerJ. 2024 Mar 5;12:e17012. doi: 10.7717/peerj.17012 (PMC10921929; doi:10.7717/peerj.17012)
Supplement: Supplemental Information 3 [file peerj-12-17012-s003.docx]

**Description of categorical data**

**Sex**: 1: Male; 2: Female.

**Statins application history** : 1: Never; 2: Intermittent administration; 3: Use statins continuously for more than one year; 4: Unknown.

**Hypertension**: 1: No; 2: Yes; 3: Unknown.

**Diabetes**: 1: No; 2: yes; 3: Unknown.

**Dyslipidemia**: 1: No; 2: Yes; 3: Unknow.

**History of stroke**: 1: No; 2: Yes; 3: Unknown.

**Smoking status**: 1: Never; 2: Former; 3: Current; 4: Unknown.

**Clinical diagnosis**: 1: NA; 2: Unstable angina pectoris (UAP); 3: Angina pectoris (AP); 4: ST-segment elevation myocardial infarction (STEMI); 5: Non-ST-segment elevation myocardial infarction (NSTEMI); 6: Unknown.

**Number of diseased vessels**: 1: 0; 2: 0.5; 3: 1; 4: 2; 5: 3.
